# Continuing the vision: engaging the Ottawa Charter for future ‘healthy public policy’

**DOI:** 10.1093/heapro/daaf178

**Published:** 2025-10-16

**Authors:** Belinda Lunnay, Frances Baum, Candace Angelo, Gemma Crawford

**Affiliations:** Research Centre for Public Health, Equity and Human Flourishing, Torrens University Australia, Wakefield Campus, 88 Wakefield Street, Adelaide, South Australia 5000, Australia; Stretton Health Equity, Stretton Institute, Napier Building, Adelaide University, North Terrace, Adelaide, South Australia 5000, Australia; Sydney School of Public Health, Faculty of Medicine and Health, The University of Sydney, A27 Fisher Road, Camperdown, New South Wales 2050, Australia; Collaboration for Evidence, Research and Impact in Public Health, Curtin School of Population Health, Curtin University, GPO Box U1987, Perth, Western Australia 6845, Australia

Celebrating 40 years of the Ottawa Charter for Health Promotion prompts reflections on its influence in Australia since the World Health Organization (WHO)’s second International Conference on Health Promotion. We reflect on the ways that ‘healthy public policy’, central to the Charter, has been implemented and understood since then. Australia hosted the conference 2 years after the Charter’s creation. The conference happened at the end of a decade dominated by HIV/AIDS. Australia was a global trailblazer in its epidemic response, centring community participation and no-blame approaches (Brown *et al*. 2014). The Hawke Government had also introduced a universal health insurance scheme—Medicare (1984) and Australia continued benefiting from the Whitlam Government’s Community Health Policy legacy. These landmark policies addressed health system shortfalls to increase service accessibility through a social model of health.

## COMMITMENT TO SOCIAL JUSTICE

The Australian government showed strong commitment to the conference, held in Adelaide, South Australia on Kaurna Country—a state with a Social Health Strategy (South Australian Health Commission 1988). Prime Minister Bob Hawke opened the conference and the Minister for Community Services and Health Dr Neal Blewett and WHO Director General, Dr Halfdan Mahler were co-chairs. Mahler (1988) stressed how public health’s history was about structural change but had been replaced by ‘behavioural victim-blaming’. His closing message remains relevant—we need healthy public policy ‘in order to protect the interests of future generations and the survival of this planet Earth’ (p. 138).

The 1988 conference confirmed the aim of healthy public policy: ‘to create a supportive environment to enable people to live healthy lives’. It reaffirmed the Alma Ata Declaration reinstating social justice as a prerequisite for health, the importance of co-operation across sectors and of people’s participation. The conference ended with accepted recommendations: supporting women’s health, enabling access to healthy food and nutrition; reducing tobacco growing and alcohol production, marketing and consumption; and creating supportive environments for health. The conference made a ‘special plea’ to link the new public health to ecology and the environment and to link the ‘developed’ world and the ‘developing’ world in ‘co-ordinated’ efforts for health.

## LESSONS ABOUT PARTICIPATION AND INCLUSION

The conference taught lessons about participation and the necessary inclusion of local people and advocacy groups. This point was foregrounded when Aboriginal women from Central Australian Aboriginal Congress (a community-controlled health organization) took centre stage in the closing ceremony to draw attention to the failure of the Australian government to fund Alukura, a women’s birthing service (Central Australian Aboriginal Congress 2021). This was critical; Australia was celebrating the Bicentennial of British invasion and coming to terms with its colonial history and impact on the health and wellbeing of Aboriginal and Torres Strait Islander peoples.

Aboriginal Community Controlled Health Services (ACCHSs) developed at the beginning of the 1970s were expanding nationally and embodied the Ottawa Charter’s call for community participation and empowerment. ACCHSs promoted a decolonizing model of health promotion and challenged mainstream systems to move beyond deficit framings and to recognize Indigenous knowledges and self-determination as central to effective and ethical policy. ACCHSs have been very effective advocates for healthy public policy.

## POLICY WINS AND CHALLENGES

Examples of Australian public policy with equity and health impact span seatbelt mandates, needle syringe programmes, tobacco packaging and nutritional labelling, to paid parental leave and decriminalizing homosexuality. Battles surrounding their implementation reveal tensions between health promotion principles and political realities. For example, the Voice to Parliament referendum defeat in 2023, following a campaign marked by racism and misinformation, highlights persistent structural barriers to meaningful policy participation by those affected.

Many initiatives (and potential new ones) remain ‘isolated’ to health portfolios rather than the systems-level action the Charter envisioned. However, comprehensive intersectoral approaches demonstrated great potential. Adoption of Health in All Policies (SA) (Kickbusch *et al*. 2008) and Healthy Cities (e.g. Noarlunga) reflect whole-of-government mandates with supportive political rhetoric (Baum *et al*. 2006). Without political will, even successful movements are difficult to sustain, evidenced by defunded community health programmes (Lewis *et al*. 2025).

## INSTITUTIONALIZING HEALTH PROMOTION

The conference (1988) coincided with unprecedented enthusiasm for health promotion Australia-wide. Professional associations and university programmes were established and accredited based on health promotion competencies reflecting Charter principles and providing an enabling environment.

Health promoting policy in Australia is buoyed by incremental increases in supportive infrastructure—health promoting agencies VicHealth and Healthway were each funded through tobacco taxation. The sector has built momentum to address climate change (e.g. Climate and Health Alliance), resulting in the Climate and Health Strategy (2021–25). Australia’s National Preventive Health Strategy (2021–30) conveys that health promotion is critically important and cost-effective (Smith *et al*. 2016).

The National Aboriginal and Torres Strait Islander Health Plan (updated 2021) situates culture as a determinant of health, aligning closely with the Charter’s emphasis on enabling environments. Aboriginal health promotion has consistently demonstrated that community governance and accountability are core to designing and sustaining healthy public policy. Aboriginal and Torres Strait Islander health promotion became institutionalized through dedicated Aboriginal health units and cultural safety training—re-orienting health services, which is a key principle of the Charter. Australia is also achieving growing recognition of Indigenous data sovereignty in research and policy.

Victoria is the first Australian jurisdiction to establish a Treaty with Aboriginal and Torres Strait Islander peoples. The Preventive Health SA Act 2024 permanently built into legislation a principle ‘to improve collaboration between government agencies’. Western Australia has established the first Minister for Preventive Health. Such momentum signals opportunities for national support for legislature and joined-up action (Smith *et al*. 2016).

## THE OTTAWA CHARTER—INTO THE NEXT 40 YEARS

The Charter is a touchstone for the value of healthy public policy in Australia in interlinked ways.

### Complex understanding of healthy public policy

Australia needs investment in healthy public policy reflecting real-world complexity that is not ‘coercive’ but emancipatory (Carey *et al*. 2015). The Charter centres shared power, empowerment, and participation; these must be recognized in new policy. Ideologies which promote minimal government intervention do not recognize the complex, systemic analysis of ‘problems’ and nuanced, contextual, and equity-driven requirements of ‘solutions’.

### Developing evidence to inform healthy public policy

The Charter provides a theory of action that can evaluate impact (Thomas *et al*. 2025), including on reducing inequity. It provides a conceptual frame founded on social-ecological (Bronfenbrenner 1979) and salutogenic (Antonovsky 1996) theory that can guide research to inform policy (Fry and Zask 2017). These ideas complement approaches which prioritize biomedicine in public health research informing policy (Lunnay and Foley 2024). This is not to undermine traditional scientific methods but elevate community experience as a valid form of evidence (Windle *et al*. 2025). It includes conscious efforts towards decolonizing ways of knowing, doing, and being; where lived experience of structural contexts are acknowledged, including the complex intersections between determinants of health.

### Responding to corporate appropriation of health promotion terminology

A major issue in the coming decades will be responding to complex commercial and digital determinants of health (Kickbusch and Holly 2023, Thomas *et al*. 2024). Initiatives that reflect the corporate capture of the Ottawa Charter for Health Promotion terminology and action, like BUPA’s ‘Building Healthy Cities’ Campaign, which appropriates health promotion terminology to promote corporate goals. Such campaigns conflict with Charter values as they promote universal health messaging while profiting from privatized care and do not promote equity. Commercial interests pose persistent policymaking challenges and orienting economies towards wellbeing requires political traction for systems-level change (Crawford and Trebeck 2025).

### Supporting self-determination and sovereignty

To achieve equity through healthy public policy, the next 40 years must see the strengthening of Aboriginal and Torres Strait Islander leadership—particularly as Australia progresses with Treaty processes and grapples with the unfinished business of reconciliation. Embedding the Charter’s principles in future public policy involves Indigenous-led frameworks which mediate cultural determinants of health for equity and enable community control. This means not only recognizing Aboriginal and Torres Strait Islander peoples as priority populations but elevating their knowledges, governance structures, and rights within policymaking processes.

## CALL TO ACTION—ACHIEVING THE ‘HEALTH FOR ALL’ DREAM

The Ottawa Charter for Health Promotion remains relevant now and into the future, providing policy actors with a framework to navigate changing environments and bring about systems-level change informed by community engagement. At the Adelaide Conference, Health Minister Neal Blewett called for considerable structural change (Blewett 1988) to achieve the ‘Health for All’ dream. Re-engaging with the ‘Adelaide Recommendations’ can garner policy linked to this dream and continue the commitment.

## Data Availability

Not applicable.
